# Telemedicine in Obstetrics: New Era, New Atitudes

**DOI:** 10.1055/s-0040-1715145

**Published:** 2020-07

**Authors:** Sue Yazaki Sun, Cristina Aparecida Falbo Guazzelli, Jorge Francisco Kuhn dos Santos, Cláudia Galindo Novoa, Rosiane Mattar

**Affiliations:** 1 Department of Obstetrics, Escola Paulista de Medicina, Universidade Federal de São Paulo, São Paulo, SP, Brazil

The COVID-19 outbreak started in December 2019 in China, and spread throughout the world as a big threat since then. On March 11th, the World Health Organization (WHO) declared it a pandemic. In Brazil, the first case was diagnosed on February 26th on a man that had recently returned to Brazil from Italy, and on March 13th, Brazil diagnosed its first case of community infection. Followed by that, on March 20th, the Brazilian Health Ministry intensified the national efforts to control and prevent the fast spread of the disease, issuing ordinance under number 454,^1^ which recommends social distancing measures. The elderly – specifically the population over 60 years of age – were considered a group of risk, and were advised to limit their activities to what are considered essential services and to avoid crowded spaces, such as cultural and scientific events. In accordance with the national recommendation, the state of São Paulo declared quarantine on March 24th, under decree number 6,4881.^2^

In this new national scenario, the Brazilian Federal Council of Medicine, through official notice number 1,756/2020, issued on March 19th, communicated the Health Ministry that it recognized the possibility and ethics of the use telemedicine, as an exception, during the battle against the transmission of COVID-19, using teleorientation, telemonitoring and teleinterconsult. On April 15th, the Federal Government enacted law number 13,989,^3^ which regulates the use of telemedicine during the COVID-19 crisis as an emergency measure. Moreover, the law defines telemedicine as medical practice mediated by technology to promote health, research, patient care and prevention of diseases.

For decades, the Department of Obstetrics at Escola Paulista de Medicina, Universidade Federal de São Paulo, has been promoting daily rounds. These meetings are held at Hospital São Paulo, a tertiary service that is a reference in high-risk obstetrics cases. All cases of patients hospitalized and cared for by the Obstetrics team are discussed with professors, medical staff, residents and students – with almost twenty people in the room per meeting.

With the advent of the pandemic, this model of rounds had to be significantly modified. Numerous professors – particularly those most experienced – were advised to avoid going to the hospital because they were older than 60 years of age. Therefore, our department had to remodel its assistance and adequate it to the previously-discussed legal terms. In this new model, the daily rounds are now held using the Google Meet platform, and they are considered teleguidence or teleinterconsultation,^4^ with the residents and medical staff providing care at the hospital, and the professors, remotely from their residences. The decisions that are made during the meetings are recorded in the electronic medical records of the patients by the residents, and, simultaneously, one of the professors accesses the same records, remotely, from their residence, to validate the decisions.

The social isolation imposed by the pandemic has stimulated the use of technology as a fundamental tool not only in our context, but also around the world – as illustrated in a recent editorial^5^ about use of telemedicine in American universities. In our daily routine, it has saved time previously used to commute to the hospital, lowered the consumption of eletricity due to the lower use of the elevator, and lowered expenses with parking tickets etc. Moreover, the virtual rounds broke geographical barriers and enabled even more physicians in our department to participate, since it is now easier for them to adjust their personal schedules to the schedule of the rounds. Therefore, this new model enabled the dissemination of knowledge and experience beyond the University's gates.

Our experience during this pandemic has shown that teleguidance and telesupervision are possible. Moreover, if they are performed exchanging information through safe virtual platforms, and if the notes on the medical records are inserted by residents in-site and by the professors remotely, it is possible to guarantee privacy and co-responsibility in medical assistance.

With the pandemic and subsequent social isolation, other activities that had been previously suspended were resumed virtually. Now, scientific meetings with invited guests, administrative meetings and academic classes have been made possible with the use of technology. It is clear that telemedicine is viable and advisable for assistance and educational purposes in Obstetrics, and that it should be used strictly according to the regulation norms. In our service, this tool has enabled us to mantain a high level of obstetric care, and we hope that future national laws make it possible for us to use telemedicine after the COVID-19 crisis.
